# Evidence for a Specific Association Between Sustained Attention and Gait Speed in Middle-to-Older-Aged Adults

**DOI:** 10.1093/geroni/igab046.2647

**Published:** 2021-12-17

**Authors:** Courtney Aul, Hannah Park, Joseph DeGutis, On-Yee Lo, Victoria Poole, Jonathan Bean, Elizabeth Leritz, Michael Esterman

**Affiliations:** 1 VA Boston Healthcare System, Boston, Massachusetts, United States; 2 Brandeis University, Waltham, Massachusetts, United States; 3 VA Boston Healthcare System/Harvard Medical School, Boston, Massachusetts, United States; 4 Hebrew SeniorLife/Harvard Medical School, Boston, Massachusetts, United States; 5 Rush University, Chicago, Illinois, United States; 6 VA Boston Healthcare System, VA Boston Healthcare System, Massachusetts, United States; 7 VA Boston Healthcare System/BU School of Medicine, Boston, Massachusetts, United States

## Abstract

Although cognitive decline has previously been associated with mobility limitations and frailty, the relationship between sustained attention and gait speed is incompletely characterized. To better quantify the specificity of the sustained attention and gait speed association, we examined the extent to which this relationship is unique rather than accounted for by executive functioning and physical health characteristics. 58 middle-to-older-aged community-dwelling adults without overt illness or diseases (45-90 years old, 21 females) participated in the study. Each participant completed a 4-meter gait speed assessment and validated neuropsychological tests to examine various domains of executive functions including working memory (i.e., Digit Span), inhibitory control (i.e., Stroop Color Word Test), and task switching (i.e., Trail Making Test). Multiple physical and vascular risk factors were also evaluated. Sustained attention was assessed using the gradual onset continuous performance task (gradCPT), a well validated go/no-go sustained attention task. A series of linear regression models were created to examine how different aspects of cognition, including sustained attention and traditional measures of executive functioning, related to gait speed while controlling for a variety of physical and vascular risk factors. Among all predictors, gradCPT accuracy explained the most variance in gait speed (R2 = 0.21, p < 0.001) and was the only significant predictor (β = 0.36, p = 0.01) when accounting for executive functioning and other physical and vascular risk factors. The present results indicate that sustained attention may be uniquely sensitive and mechanistically linked to mobility limitations in middle-to-older adults.

